# Role of the WHO Surgical Safety Checklist in Reducing Morbidity and Mortality Among Obstetrics and Gynecology Patients Undergoing Surgery: A Prospective Comparative Study

**DOI:** 10.7759/cureus.60775

**Published:** 2024-05-21

**Authors:** Amrita Amrita, Jaya Kumari, Archana Sinha, Akanksha Singh, Neeru Goel, Poonam Poonam, Mumtaz Hussain

**Affiliations:** 1 Department of Obstetrics and Gynaecology, Indira Gandhi Institute of Medical Sciences, Patna, IND; 2 Department of Anaesthesiology, Indira Gandhi Institute of Medical Sciences, Patna, IND

**Keywords:** surgical site infection, sepsis, postoperative complications, surgery-related complications, obstetrics, gynecology, world health organization surgical safety checklist, patient safety

## Abstract

Background:As surgery is an essential aspect of healthcare around the globe, it is necessary to consider complications related to it. Therefore, this study was conducted to evaluate the impact of the World Health Organization Surgical Safety Checklist (WHO SSC) on reducing the incidence of postoperative complications

Methods*: *This single-center, prospective, comparative study was conducted at the Department of Gynecology and Obstetrics in a government hospital in Patna, Bihar. To assess the efficacy of the WHO SSC, the patients were divided into two groups, in which one group undergoing surgery was assessed with the checklist, and the other group was not. The rates of surgery-related complications were then compared in both groups.

Results:Our results showed a reduction in surgery-related complications in patients assessed with the WHO SSC. No statistically significant difference in duration of surgery was found between the groups. However, a statistically significant difference was observed in the rates of surgery-related complications between groups, especially in sepsis (p*=*0.0009), hemorrhage (p*<*0.0001), and infection at the site of surgery (p*<*0.0001). Mortality rates were not affected by the use of the SSC.

Conclusion:The WHO SSC is a simple yet effective tool for reducing postoperative complications by improving communication between the various team members working in the operation theatre, although it has no effect on reducing mortality. Further research is needed to enhance its successful implementation and ensure its sustained use.

## Introduction

Over 234 million surgeries are estimated to be performed annually, rendering surgical care an essential aspect of healthcare throughout the globe [1]. Although surgery can save the life or limb of an individual, there remains a significant risk of implications, including hemorrhage, various infections, sepsis, and inflammation [2-4]. Therefore, patients who undergo surgical procedures often face immense risks, but perioperative surgical safety checklists can help to reduce surgical site infections (SSIs), disruptions of wound healing, and sepsis [5].

In many parts of the world, the risk of surgical complications is not adequately known; however, the reported crude mortality rate after major surgery is 0.5-5%, and complications after inpatient operations occur in up to 25% of patients [1]. It has been observed that the complication rate is much higher in developing countries, including India [6-9]. The essential objectives of safe surgery include site accuracy, safe anesthesia administration, managing airway problems and hemorrhage, avoiding known allergies, minimizing the risk of surgical site infection, effective communication within the surgical team, and routine surveillance of surgical outcomes.

In 2008, the World Health Organization (WHO) published recommendations including suggested actions for ensuring the safety of surgical patients worldwide [10]. The 19-item WHO Surgical Safety Checklist (SSC) was implemented to increase perioperative safety, as it demonstrated lower rates of perioperative mortality and complications in a variety of healthcare settings [11]. Usage of the SSC can lead to the development of a safer approach among surgical staff [12].

The WHO SSC is intended to be globally applicable to ensure patient safety during surgery. It has been applied in a variety of surgical settings, including ambulatory surgery, endoscopy, and labor and delivery [13]. This study was performed to evaluate the effect of implementing the WHO SSC on reducing various co-morbidities and mortality among surgical patients in the Department of Obstetrics and Gynecology at a tertiary-level government hospital in India.

## Materials and methods

Study design

This was a single-centric, prospective, comparative study performed at the Department of Obstetrics and Gynecology of a government hospital, Indira Gandhi Institute of Medical Sciences, in Patna, Bihar. The study duration was two years. Ethical approval to conduct the study was given by the Institutional Ethics Committee of Indira Gandhi Institute of Medical Sciences, Patna (approval number: 40/IEC/IGIMS/2021).

Overall, 400 patients undergoing routine gynecological surgeries in the Department of Obstetrics and Gynecology were enrolled in the study. The participants were divided equally into two groups. The SSC was used in Group 1 (n=200) while it was not used in Group 2 (n=200). Informed consent for participation in the study was obtained from patients before initiating surgery. Before the initiation of the study, its details and purpose were explained to the participants.

Sample size calculation

Based on previous literature [14], we calculated the sample size for this study using the following formulas: n1=(σ2 + σ2k) (Z1-α/2 + Z1-β)/d2, with a statistical power of 95% and a type-2 error of 5%; and n2=K*n1, where d is the difference between two means, σ2 is the variance, k is the ratio of sample size between groups 1and 2, α is the probability of type-1 errors, β is the probability of type-2 errors, and Z is the critical value given for α and β taken through estimation of powers. According to the calculated sample size, 400 patients were enrolled in the study and then divided equally into two groups (i.e., 200 in Group 1 and 200 in Group 2) [3].

Data collection

**Figure 1:**
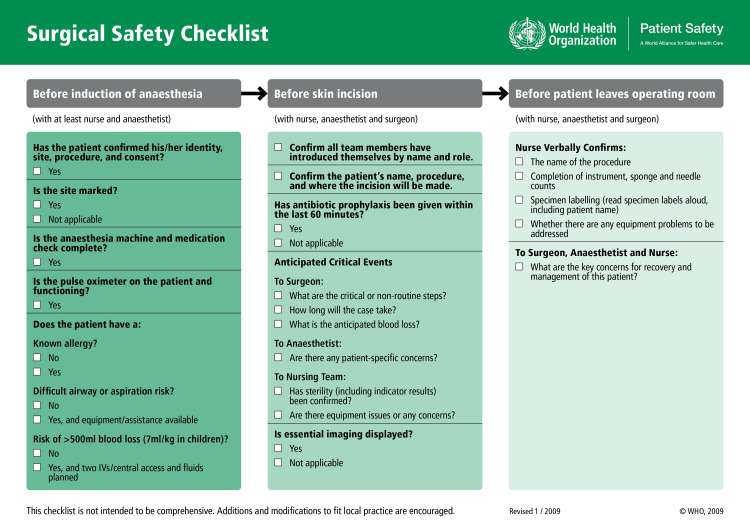
WHO Surgical Safety Checklist Image Source: WHO Guidelines for Safe Surgery 2009 [11]. Reproduced with permission from the World Health Organization.

In Group 1, the WHO SSC (Figure 1) was filled by the resident on duty in three phases: (i) before the induction of anesthesia, (ii) before skin incision, and (iii) before the patient left the operation theatre. The resident on duty confirmed that the anesthetist, surgeon, and nursing team had completed the listed tasks in the SSC before proceeding with the surgery and exit. Data on patient demographics were collected.

Study outcomes

The study outcomes were measured by surgical residents who did not participate in the study to avoid bias. Complications were assessed in the patients following surgery such as sepsis (based on systemic inflammatory response syndrome (SIRS) criteria), SSI or open-wound infection, and hemorrhage (defined as blood loss of more than 500 ml). Mortality rate was also assessed during further follow-ups till one month after surgery. Finally, the impact of WHO SSC documentation on patient outcomes was examined.

Statistical analysis

The data comprised both continuous and categorical variables. Quantitative variables were expressed as mean ± standard deviation (SD), frequency (percentage), or frequency. Statistical homogeneity was evaluated using Pearson’s chi-squared test. ANOVA tests were performed to analyze variance within groups, and Tukey’s test was used for post-hoc analysis. Two-tailed p-values were used in all statistical tests, where p<0.05 was considered statistically significant. All statistical analyses were performed using IBM SPSS Statistics for Windows, Version 26.0 (Released 2019; IBM Corp., Armonk, New York, United States).

## Results

The demographics and health status of the patients were assessed individually, and then both groups were compared to assess the impact of the WHO SSC. Table 1 summarizes the age characteristics of the two groups. Most of the patients in both groups were >40 years of age. Very few patients were <40 years of age. Differences between most age groups were not statistically significant, with the exception being the 50-60 age group.

**Table 1:** Distribution of participants in the different age groups. Data presented as frequencies (percentages) *p-values considered significant at <0.05 (two-tailed; Pearson’s chi-square test)

Age (years)	Group 1 (N=200), n (%)	Group 2 (N=200), n (%)	p-value
<30	31 (15.5%)	16 (08%)	2.39
30–40	38 (19%)	41 (20.1%)	0.06
40–50	67 (33.5%)	74 (37%)	0.17
50–60	59 (29.5%)	62 (31%)	0.04*
>60	05 (2.5%)	07 (3.5%)	0.17

Table 2 summarizes the distribution of the time taken to complete the surgery. Most surgeries took place in less than one hour, and very few lasted three hours. Our results showed that there was no statistically significant difference between the groups in terms of duration of surgery. So, it could be stated that in this study there is no correlation between the duration of surgery among those who were assessed using the WHO SSC and those who weren’t.

**Table 2:** Duration of surgery in both groups. Data presented as frequencies (number of patients) *p-values considered significant at <0.05 (two-tailed; Pearson’s chi-squared test)

Duration of surgery (hours)	Group 1, n	Group 2, n	p-value
≤1	102	112	0.23
1–2	65	59	0.15
≥2 to 3	33	29	0.13
Total	200	200	

As seen in Table 3, statistically significant differences were observed in the rates of most surgery-related complications (e.g., sepsis, hemorrhage, and SSI), except mortality, which was not significant among the groups. Sepsis and hemorrhage were the most commonly occurring complications related to surgery. However, surgery-related complications were higher in the group in which the WHO SSC was not used (Group 2). Differences in the rates of sepsis, hemorrhage, and SSI were especially significant (p-value<0.0001 in sum), indicating the positive impact of the WHO SSC on complication rates. Mortality rates were largely similar when compared in both groups, indicating no association with WHO SSC use.

**Table 3:** Complications related to surgery. Data presented as means±standard deviations *p-values considered significant at <0.05 (two-tailed; ANOVA test used for comparison of surgery-related complications in different groups, and Tukey’s test used further for post-hoc analysis) SIRS: systemic inflammatory response syndrome; SSI: surgical site infection; ER: emergency room

Surgery-related complication	Group 1 (N=200), mean±SD	Group 2 (N=200), mean±SD	p-value
Sepsis (SIRS criteria) [15]	19.23±8.1	25.3±11.1	0.0009*
Hemorrhage (Blood loss >500 ml) [16]	14.72±6.3	29±16.2	<0.0001*
SSI (Evaluated on ER visits)	9.03±2.8	14.16±6.96	<0.0001*
Mortality rate (including both intra-operative as well as within a month of surgery)	3.5±1.4	3.9±2.7	0.063

## Discussion

Our results showed that the use of the WHO SSC had a positive effect on patients who underwent surgery. Post-operative complications such as sepsis, hemorrhage, SSI, and mortality were less prevalent in those who were assessed with the WHO SSC, indicating the strong positive effect of the intervention. The rates of surgery-related complications were higher in the group where the WHO SSC was not implied. Mortality rates were not significantly affected by the use of the WHO SSC. Finally, the use of the WHO SSC resulted in no significant difference in the duration of surgery between the groups. The duration of surgery was considered an outcome variable, as studies have shown the association of increased operative time with increased complication rates in gynecological surgeries [17]. These results highlight the importance of the implementation of the WHO SSC before surgery. Finally, no significant difference was observed between different age groups. Our results agreed with those of a similar study, which found that implementation of the WHO SSC resulted in the reduction of adverse events in patients who underwent surgery [18].

In a similar study conducted in eight cities, the assumption that a surgical checklist would have a significant impact on patients undergoing surgery was verified, as the results showed that usage of the checklist led to reduced complications [12]. In an observational study done to assess the effect of the WHO SSC on patient outcomes and documentation, the implementation of the checklist resulted in improved outcomes [18]. In a prospective, non-randomized, comparative survey done to compare the effect of implementing the WHO SSC before and after surgery in an oropharyngeal setting, the checklist had a favorable impact on factors related to patient safety [13]. In a prospective, observational study including 352 participants, trained research nurses implemented the WHO SSC and found that the implementation of the checklist was suboptimal within the scope of improvement of safety outcomes, it also showed that compliance with all items on the checklist and active participation by all team members are crucial for successful implementation of the checklist [19]. However, in another study, the Hawthorne effect was attributed to improving surgical outcomes due to subjects’ knowledge of being observed [20]. Meanwhile, the WHO SSC template was not intended to be comprehensive [13].

As surgery is a complicated task involving many people taking part in the patient care chain, good communication is critical to ensure the safe care of patients and proper team functioning. Even in the duration of procedures or evaluating checklists, there must be adequate communication and collaboration among the team members to achieve patient safety. To accomplish this goal, the WHO SSC as a tool helps all team members to communicate with each other and adequately express any concerns. An observational study done by Pugel et al. also demonstrated that WHO SSC implementation helped to enhance communication and reduce surgical complications [21]. Therefore, the use of such checklists represents a simple and inexpensive method to reduce surgery-related complications.

The purpose of the WHO SSC is to raise awareness among the patients and surgical teams about the execution of safety processes to reduce surgery-related complications, as indicated by our analysis. Our results aligned with most of the research already done on the WHO SSC. This research may provide a beneficial approach for the usage of a safety checklist in patients undergoing surgical procedures.

Study limitations

The major limitation of the study was the Hawthorne effect, leading to better outcomes in the group, where SSC was used (Group 1). The surgeons, anesthetists, and nurses would have been more attentive in Group 1 under the impression of being under scrutiny, leading to fewer complications. In addition, as the study took place at a single center, data were not collected from other similar sites, which limited the evaluation of the effectiveness of the checklist.

## Conclusions

The WHO SSC is a simple, inexpensive, and readily available tool, which can be used in an operating room to reduce complications related to surgery. It ensures patient safety by improving communication among team members involved in surgery. So, introducing this in hospitals where it is still not being used and ensuring compliance can prove crucial for reducing complications associated with surgeries. A multicentre study is needed to assess the barriers to implementation and compliance in the use of the SSC in India.
